# Structural Applications of Thermal Insulation Alkali Activated Materials with Reduced Graphene Oxide

**DOI:** 10.3390/ma13051052

**Published:** 2020-02-27

**Authors:** Wu-Jian Long, Can Lin, Xiao-Wen Tan, Jie-Lin Tao, Tao-Hua Ye, Qi-Ling Luo

**Affiliations:** Guangdong Provincial Key Laboratory of Durability for Marine Civil Engineering, Shenzhen Durability Center for Civil Engineering, College of Civil and Transportation Engineering, Shenzhen University, Shenzhen 518060, China; longwj@szu.edu.cn (W.-J.L.); lincan2018@email.szu.edu.cn (C.L.); tanxiaowen2017@email.szu.edu.cn (X.-W.T.); taojielin2018@email.szu.edu.cn (J.-L.T.); yetaohua2017@email.szu.edu.cn (T.-H.Y.)

**Keywords:** alkali activated materials, waste expanded polystyrene beads, reduced graphene oxide, thermal insulation, mechanical properties

## Abstract

Development of low thermal conductivity and high strength building materials is an emerging strategy to solve the heavy energy consumption of buildings. This study develops sustainable alkali activated materials (AAMs) for structural members from waste expanded polystyrene (EPS) beads and reduced graphene oxide (rGO) to simultaneously meet the thermal insulation and mechanical requirements of building energy conservation. It was found that the thermal conductivity of AAMs with 80 vol.% EPS and 0.04 wt.% rGO (E8–G4) decreased by 74% compared to the AAMs without EPS and rGO (E0). The 28-day compressive and flexural strengths of E8–G4 increased by 29.8% and 26.5% with the addition of 80 vol.% EPS and 0.04 wt.% rGO, compared to the sample with 80 vol.% EPS without rGO (E8). In terms of compressive strength, thermal conductivity, and cost, the efficiency index of E8–G4 was higher than those of other materials. A building model made from AAMs was designed using building information modeling (BIM) tools to simulate energy consumption, and 31.78% of total energy consumption (including heating and cooling) was saved in the building operation period in Harbin City, China. Hence, AAMs made of waste EPS beads and rGO can realize the structural and functional integrated application in the future.

## 1. Introduction

Building industry consumes vast amounts of energy and generates considerable CO_2_ emissions annually [1,2]. According to reports, more than 30% of the annual global primary energy and 70% of electricity demand is generated by buildings, which results in a huge energy expenditure [3,4,5]. Heating, ventilation, and air conditioning (HVAC) of buildings is one of the major contributors for these figures [6,7]. Besides modifications to thermal systems to reduce the energy consumption of heating and cooling in buildings, researchers are turning toward insulation materials that can be incorporated into construction materials [8,9,10]. To this end, researchers have used expanded polystyrene (EPS) waste in thermal insulation cement composites for its insulation properties and environmental-friendliness [11]. EPS is widely used to make insulation and packaging materials due to its dimensional stability, versatility, and economical efficiency [12,13]. An individual EPS bead is approximately spherical and contains 2% polystyrene and 98% air [14]. In 2016, 6200 kt of EPS were manufactured. Only 30% of the EPS were recycled after use. The rest were either disposed in landfills or incinerated, which causes serious environmental pollution [13,15]. Waste EPS beads have significantly lower density (0.028 kg/m^3^) and thermal conductivity (TC, 0.042 W/m·K) than other lightweight aggregates, such as crumb rubber (1.05–1.20 kg/m^3^) and shale aggregates (0.6–0.7 kg/m^3^) [16,17,18]. Existing EPS waste recycling techniques often use hazardous solvents, which contributes further to pollution in other ways (e.g., air or water pollution) [19,20]. To solve this, the insulation properties of EPS waste and their applications in the construction industry have been studied.

Some studies have partially replaced mineral aggregates in concrete with waste EPS beads to improve building insulation properties, which reduces the amount of waste EPS beads discarded into landfills [20,21]. However, previous studies focused mainly on wall claddings, board, partitions, and other non-structural elements [11,22,23,24,25,26]. Thermal conductivity, density, and compressive strength of EPS beads as reported in previous literatures are summarized in Table 1 [23,27,28,29,30,31,32,33,34,35]. From Table 1, EPS beads improve the thermal insulation of concrete when serving as lightweight aggregates, but reduce its strength as the content increases. In general, insulating composites cannot meet the compressive strength requirements for structural applications established by the American Concrete Institute (40 MPa, ACI 213R-03 [36]). However, Chung et al. reported a satisfactory strength in concrete upon addition of EPS beads [28]. Chung also reported a TC of 1.73–1.79 W/m·K, which is higher than that of conventional insulation concrete (less than 1.0 W/m·K) [28,37]. Consequently, a balance among strength, insulation capacity, and cost is key for developing a novel and sustainable insulating material for structural applications.

In addition to energy consumption and waste recycling, CO_2_ emissions reduction is an important goal for the building and construction industry. To this end, some studies have recognized alkali activated materials (AAMs) as a reasonable alternative to Ordinary Portland Cement (OPC) and reducing CO_2_ emissions [38,39]. Generally, AAMs do not only improve the environmental benefit, but also has higher strength than OPC [38]. Although AAMs bolster cement composites containing waste EPS beads, the strength needs to be enhanced much more to meet the structural requirements. In this study, to enhance the mechanical properties of composites, this study optimizes the AAMs with nanomaterials. Nanomaterials have been widely applied in concrete to improve its mechanical performance [40,41,42,43]. Particularly, reduced graphene oxide (rGO) is used in construction for its large specific surface area, favorable mechanical strength, and high toughness [44,45,46,47]. In addition to this superior performance, rGO can effectively disperse in the highly alkaline environment of AAMs matrix (pH ~12.5) due to electrostatic stabilization and significantly improve its mechanical properties [48,49,50].

In this study, to compare and evaluate different thermal insulation materials in terms of their environmental impact, life cycle assessment (LCA) was conducted [51]. This method facilitates the synthetic analysis of energy consumption and CO_2_ emissions of the product [52]. However, the validity of LCA relies on the accuracy of data of the project. In this vein, the application of the building information modeling (BIM) technique to the building design and operational progress can help acquire accurate real-time data [53]. In addition, BIM can assess and predict the operation performance via simulation. The combination of BIM and LCA can meaningfully assess a building’s influence on the environment throughout its lifecycle [54].

Coming to the novelty of this research, this study added waste EPS beads in AAMs (WEA, AAMs containing waste EPS) to develop an environmentally-friendly AAMs with low thermal conductivity. Furthermore, we incorporate rGO into the WEA (rGO-WEA) for enhanced compressive strength. The goal for this AAMs is structural and functional applicability. The influence of waste EPS and rGO on the mechanical and thermal-insulation properties of composites was investigated by observing the mechanical strength, thermal conductivity, and density. Furthermore, to better illustrate the interfacial transition zone and pore characteristics, the rGO-WEA microstructure was analyzed by using scanning electron microscopy (SEM). Energy consumption, CO_2_ emissions, and the cost of using rGO-WEA for the entire building were evaluated using BIM and LCA tools. The resulting rGO-WEA not only has good thermal insulation and mechanical properties to meet the requirements of structural and functional applications, but also contributes to energy conservation, CO_2_ reduction, and expenses retrenchment. In the long term, sustainable and recycled composites can give full play for the environment when applied to the structure, especially in developing countries [55], and its excellent thermal insulation and mechanical properties can also improve the quality of the structure.

## 2. Materials and Methods

### 2.1. Raw Materials

**Figure 1:**
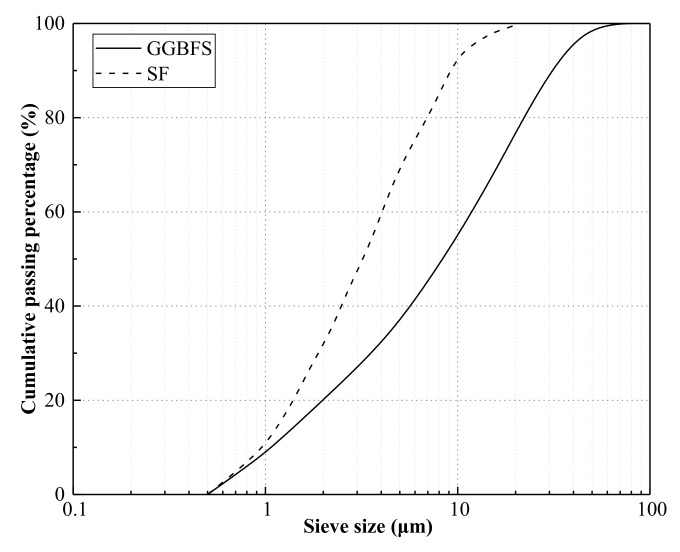
Particle size distribution of the GGBFS and SF.

Ground-granulated blast-furnace slag (GGBFS) and silica fumes (SF) used in this study were obtained from the Wuhan VCEM Technology Development Company Limited, which was conformed to Chinese National Standard GB/T 18046 and GB/T 27690 [56,57]. The specific surface areas of GGBFS and SF determined using a surface area and porosity analyzer (TriStar 3000, Micromeritics Instrument (Shanghai) Ltd., Shanghai, China) test was 853 and 1000 m^2^/kg, respectively. The mass density of GGBFS and SF determined using Lee’s bottle test was 2.9 and 1.6 g/cm^3^, respectively. In addition, the detailed chemical compositions of GGBFS and SF determined using the X-Ray Fluorescence (XRF) test is listed in Table 2. The particle size distributions of GGBFS and SF, collected by the particle size analyzer (Analysette 22 Micro Tec plus, Fritsch Beijing Scientific Instrument co. Ltd., Beijing, China) test, are shown in Figure 1.

**Figure 2:**
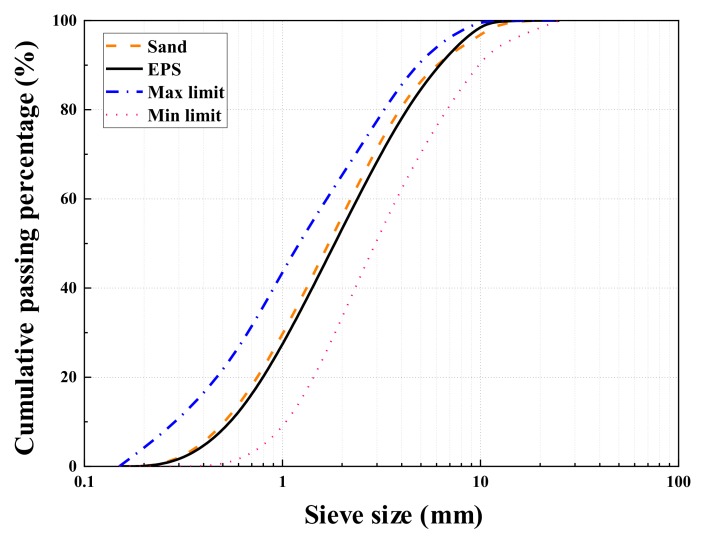
Particle size distributions of standard sand and waste EPS beads.

**Figure 3:**
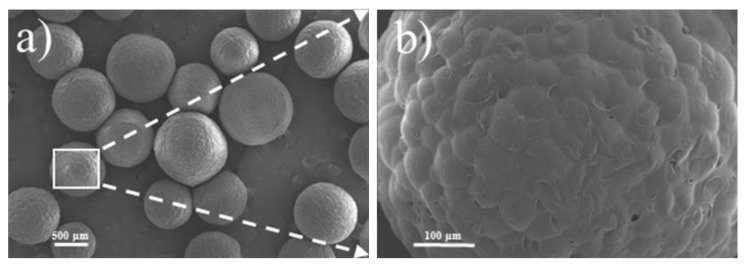
Microstructural morphology of waste EPS beads by SEM: (**a**) EPS at 20× and (**b**) EPS at 150×.

Waste EPS beads were obtained from the foam board factory. The particle size distributions of the waste EPS beads and standard sand is illustrated in Figure 2 for comparison. From the figure, the waste EPS beads and the sand have similar particle size distribution to that of standard sand and meet the maximum and minimum size distribution limits, which complies to Chinese National Standard GB/T14684 [58]. Table 3 lists the physical characteristics of the sand and waste EPS beads. The morphology of waste EPS beads is spherical with closed pores, as shown in Figure 3.

**Figure 4:**
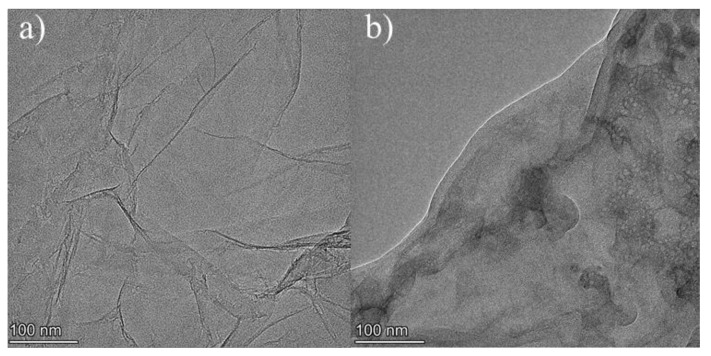
TEM images: (**a**) GO and (**b**) rGO.

The graphene oxide (GO) was prepared from graphite power by the modified Hummer’s method [59,60,61]. The product (GO) was further reduced by hot NaOH solution to obtain rGO [62,63,64]. Figure 4 shows the microstructure transmission electron microscopy (TEM) images of GO and rGO. The images reveal wrinkles on GO. The rGO, however, has fewer wrinkles due to the reduction of oxygen-containing functional groups [65]. In this study, the concentration of rGO solution was fixed at 5.0 g/L. Meanwhile, a mixture of solid NaOH and sodium silicate (Na_2_SiO_3_ with 14.3 wt.% Na_2_O, 28 wt.% SiO_2_, and 57.7 wt.% H_2_O) was utilized as the alkali activator. The modulus (molar ratio of SiO_2_ to Na_2_O) of the liquid sodium silicate (LSS) was 2.0.

### 2.2. Mix Design and Sample Preparation

Six types of samples were prepared to investigate the effect of waste EPS beads and rGO content on thermal insulation and mechanical properties of the AAMs, as shown in Table 4. The binder is a mixture of GGBFS and SF in this study. The water-to-binder ratio by mass was 0.25 for all samples. The Na_2_O concentration of the alkali activator was 4.0 wt.% relative to slag, and the modulus (SiO_2_/Na_2_O ratio) was 1.24.

**Figure 5:**
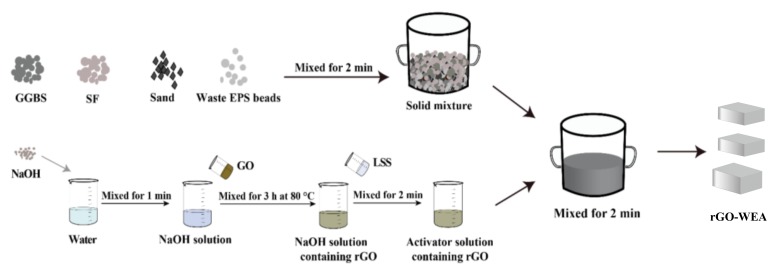
The rGO-WEA preparation process.

As shown in Figure 5, first, the solid NaOH was dissolved in H_2_O and stirred for 1 min. Then 5 g/L GO solution was added to the NaOH solution, mixing for 3 h at 80 °C to obtain rGO solution. Next, sodium silicate was added to the rGO solution and stirred uniformly for 2 min to obtain the alkali activator. Second, the waste EPS beads, SF, GGBFS, and standard sand were simultaneously poured in a mixer and mixed uniformly for 2 min into solid mixtures. The activator solution was slowly poured into the mix and stirred for another 2 min in a standard room of 20 ± 5 °C with a relatively humidity above 50%. Third, the prepared samples were discharged into standard molds and demolded after 24 h. All the samples were cured in a standard curing room of 20 ± 3 °C, with a relatively humidity above 95% for further curing. Each experiment was conducted three times, so that three samples were prepared for each mixture.

### 2.3. Test Methods

#### 2.3.1. Density Test

The density measurement was performed in accordance with ASTM C 642-13 [66]. The qualities of samples on the 28th day after 48 h in water at approximately 21 °C were weighed as the saturated mass. They were then oven-dried at 110 °C for 48 h to vaporize the water confined in the pores and weighed as the dry mass.

#### 2.3.2. Mechanical Test

To study the effect of waste EPS beads and rGO contents on the mechanical properties of rGO-WEA, the flexural strengths of three samples of 40 × 40 × 160 mm^3^ were measured. Then halves of the samples after the flexural test are used for compressive tests. The compressive and flexural strengths of rGO-WEA at the 3, 7, and 28 days were measured by Chinese National Standard GB/T 17671 [67] using a computerized electronic universal testing machine (YZH-300.10, Zhejiang Luda mechanical instrument co. Ltd., Zhejiang, China) at loading rates of 20 N/s and 2.4 kN/s, respectively. The average values were taken as the results. 

#### 2.3.3. Thermal Conductivity Test

The thermal conductivity of the samples was tested by the TC3000 thermal analyzer (XiaTech, Xian, Shanxi, China). Each test required a pair of samples to load the hot wires, and the sample was polished to minimize the effect of surface roughness. The thermal conductivity was measured by a thermal sensor installed in the gap between the two testing samples.

#### 2.3.4. Microstructural Characterization Analysis

After the mechanical tests, the broken samples were collected and placed into a drying oven at 60 °C for 12 h. The microstructure of the dry samples was observed under a Quanta FEG 250 field emission gun environmental scanning electronic microscope (SEM) (FEI Company, Hillsboro, OR, USA). The operating voltage of the instrument was set to 10 kV. The SEM images obtained had a resolution of 1536 × 1103 pixels.

#### 2.3.5. Energy Conservation Evaluation Method

**Figure 6:**
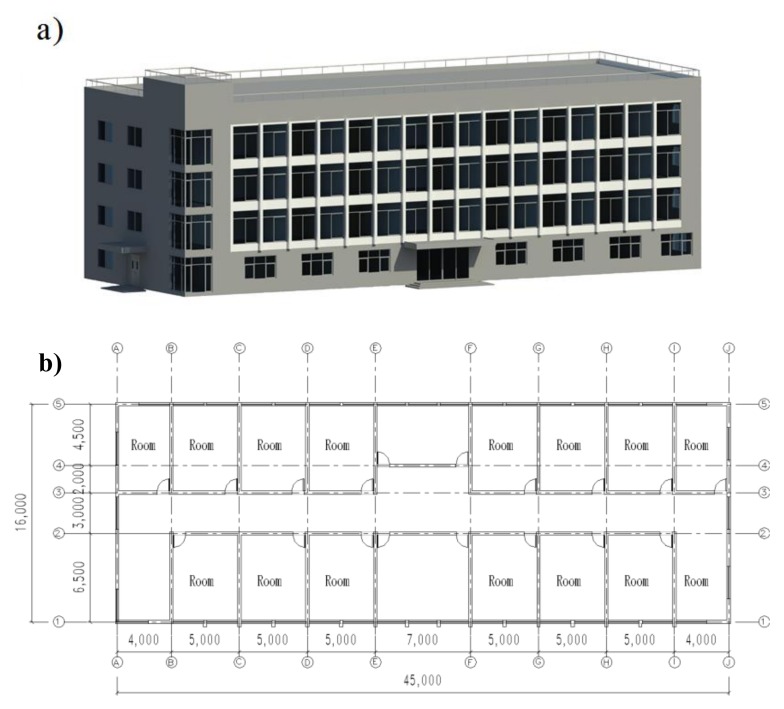
BIM model of the building: (**a**) three-dimensional view of the building and (**b**) building layout.

Materials production and building operation are critical stages in the environmental assessment. In this study, the energy conservation of constructing a building using the proposed materials were investigated by BIM. Revit software was used to build the BIM model with the building materials, spatial structure, and geographical position of the case study. As shown in Figure 6, the BIM model was designed as a four-story building with a floor area of 720 m^2^ (45 × 16 m) and a height of 3.3 m for each floor. The properties of each material in the BIM model for energy simulation is listed in Table 5.

**Figure 7:**
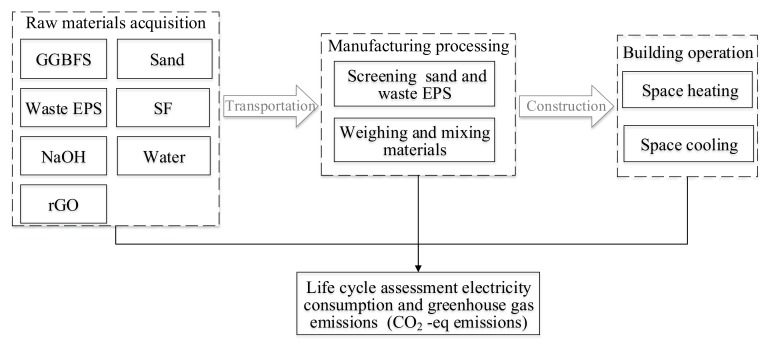
Life-cycle boundary of the life cycle assessment (LCA) evaluation.

**Figure 8:**
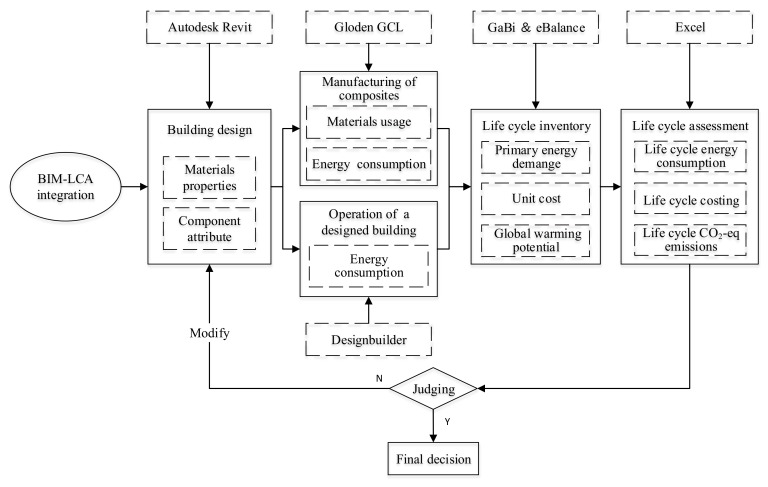
Flow chart of building information modeling (BIM) for energy conservation evaluation.

In this stage, the BIM and LCA tools were used to evaluate the potential energy consumption, CO_2_ emission, and cost of the building using the optimal mixture. Table 6 shows the basic indices obtained from the GaBi database and literatures, which indicates embodied energy (EE), embodied CO_2_ emission (ECO_2_e), and cost. These values were then utilized to calculate the relevant impact of the proposed AAMs. The life cycle boundary of the proposed composites is shown in Figure 7. In this step, BIM is treated as an information management system to acquire accurate data input for LCA. However, the calculation of the potential impact based on basic indices only cover the stage of material preparation in a building. Therefore, the BIM tool was further conducted to simulate the energy consumption of the designed building during its operation stage. The energy performance was simulated on DesignBuilder V4.5 software (using EnergyPlus dynamic simulation engine). The major inputs for the simulation are the location and weather conditions of the study area, the HVAC systems in the building, the thermal walling materials, the operating hours, and usage profile. The physical and thermal properties of the building materials were obtained from the American Society of Heating, Refrigerating and Air-Conditioning Engineers (ASHRAE) Standard [68]. The software integration process combining BIM with LCA is illustrated in Figure 8. As proposed, AAMs have few impacts on the manufacture and demolition of construction. This study mainly pays attention to the impact of the raw materials processing and building operation.

## 3. Results and Discussion

### 3.1. Density

**Figure 9:**
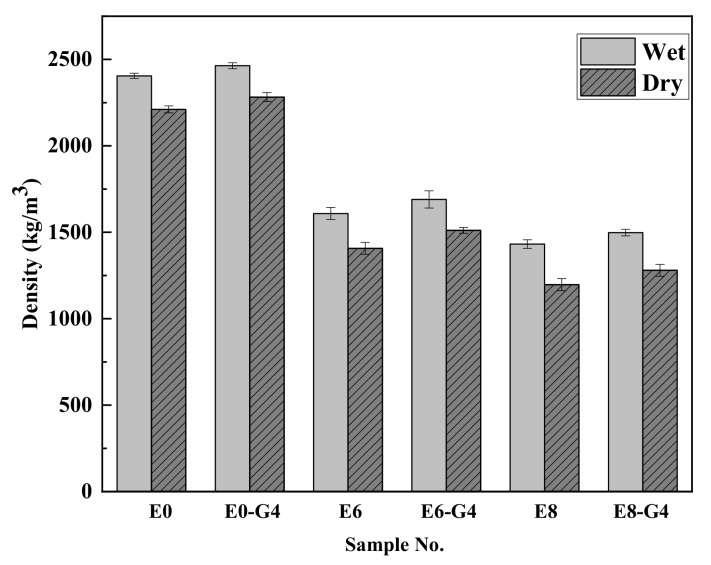
The wet and dry densities of rGO-WEA.

Density can be a significantly important factor affecting the thermal conductivity through a porous material. As expected, the wet and dry densities of rGO-WEA with waste EPS beads ranged from 1432–1689 kg/m^3^ and 1197–1511 kg/m^3^, respectively. This meets the requirement of the ASTM C 642-13 [66] for the density of lightweight, moderate-strength concrete. The addition of waste EPS beads caused a significant decrease in the density of the AAMs as compared to the presence of sand. As shown in Figure 9, the dry densities of E6–G4 and E8–G4 decreased by 31.6% and 42.1%, respectively, when compared with E0. This result was due to the increase of low-density waste EPS (28 kg/m^3^). Note that the addition of rGO slightly accelerated the alkali reaction, which leads to more reaction products and increases the density of rGO-WEA [46,48]. However, the rGO content adopted in this study was relatively low (0.04 wt.%). Therefore, the increase in wet and dry densities of rGO-WEA were not significant. For instance, the wet and dry density of E8–G4 were 1498 kg/m^3^ and 1280 kg/m^3^, respectively. This was only 4.6% and 6.7% higher than the 1432 kg/m^3^ and 1197 kg/m^3^ of E8.

### 3.2. Mechanical Properties

**Figure 10:**
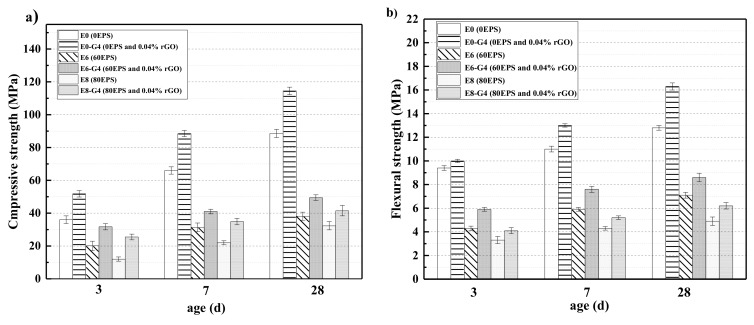
Mechanical strengths of rGO-WEA sample at different curing ages: (**a**) compressive strength and (**b**) flexural strength.

The effects of waste EPS beads and rGO contents on the compressive and flexural strengths of each sample at 3, 7, and 28 days are shown in Figure 10a,b, respectively. As illustrated in Figure 10a, the compressive strength of each sample is clearly increased by extending the curing age. However, the compressive strength clearly decreased as the volume of the waste EPS beads increased. The 28th day compressive strength of E6 and E8 decreased sharply by 56.8% and 64.0%, respectively, when compared to E0. Furthermore, a similar trend is seen with flexural strength. From Figure 10b, with the addition of 60 and 80 vol.% of waste EPS beads, the decrease in the 28th day flexural strength from E0 were 55.1% and 68.9%, respectively. This decrease in strength is primarily due to the low density and poor mechanical properties of waste EPS beads. Waste EPS beads do not contribute toward resisting the external deformation and produce greater strain under the same loading conditions. The present finding is consistent with those reached by previous works [21,23,69].

However, when compared with E8, the compressive and flexural strengths of E8–G4 starkly increased by 29.8% and 26.5%, respectively, with the addition of rGO. A similar trend was observed for the AAMs made of 60 vol.% waste EPS beads with increasing rGO content. The plausible explanation for this trend is that rGO accelerates the hydration degree, and, consequently, enhances the mechanical strength [46]. Moreover, the wrinkled surface of rGO contributes to the strong bonding state between rGO and its AAMs matrix. This strong interfacial bonding further enhances the strength because rGO effectively dissipates the stress [50,70,71].

In addition, rGO works well in the alkaline environment of concrete, which prevents the AAMs from weakening the concrete’s performance. Therefore, although waste EPS beads deteriorates the compressive strength of the AAMs, rGO reverses this trend and significantly increases the compressive strength of WEA. Note that the 28th-day compressive strength of E6–G4 and E8–G4 are 49.4 MPa and 41.5 MPa, respectively, which meets the minimum 28 d compressive strength for structural applications, according to ACI 213R-03 [36]. This proves that rGO contributes effectively to WEA applications in structural components.

### 3.3. Thermal Conductivity

**Figure 11:**
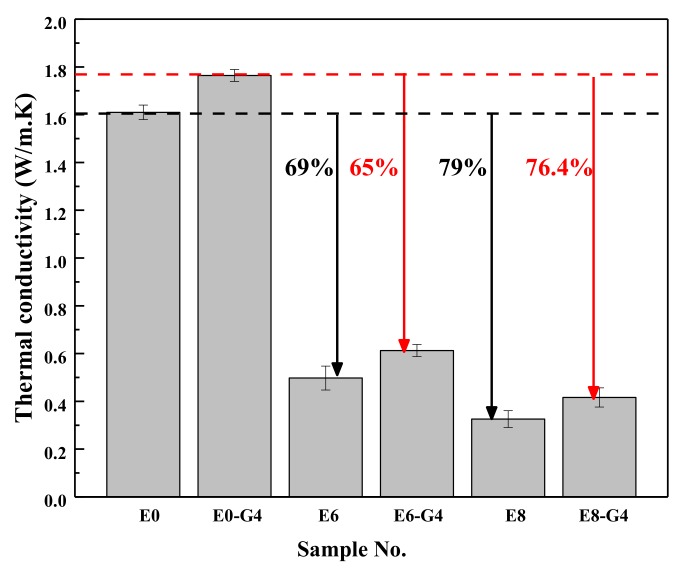
Thermal conductivity of rGO-WEA.

Thermal conductivity of material is the quantity of heat transmitted through a unit thickness in a direction perpendicular to a surface of unit area due to a unit temperature gradient under given conditions [72]. Figure 11 plots the TC of composites with different rGO and waste EPS beads content. The results of the TC test reveal a downward trend against the increase of waste EPS beads content. For example, the addition of 80 vol.% waste EPS beads contributes to 79% reduction in TC, from 1.612 W/m·K of E0 to 0.332 W/m·K of E8, relative to the specimen without EPS. The results also prove that waste EPS can effectively improve the thermal insulation of composites. It was compared with or was even lower than the data of the mortars or concretes with different lightweight aggregates such as expanded shale, pumice, expanded clay, expanded perlite, and rubber [73,74,75]. Figure 11 also reveals a slight increase in the TC with the addition of rGO. However, a mere addition of 0.04 wt.% of rGO to E8–G4 can reduce the TC by 76.4% compared to E0–G4 (1.761 W/m·K). This proves that the increase in TC caused by the addition of rGO is insignificant compared with the reduction of TC due to the addition of waste EPS beads.

The decrease of the TC with density follows a mostly linear trend. This agrees with other research studies [74,76,77]. This decrease in thermal conduction is due to the increasing number of voids being introduced by the low-density waste EPS beads. Consequently, replacing sand with waste EPS beads reduced the TC of the AAMs as a result of the porous structural aggregates used.

### 3.4. Microstructural Analysis

**Figure 12:**
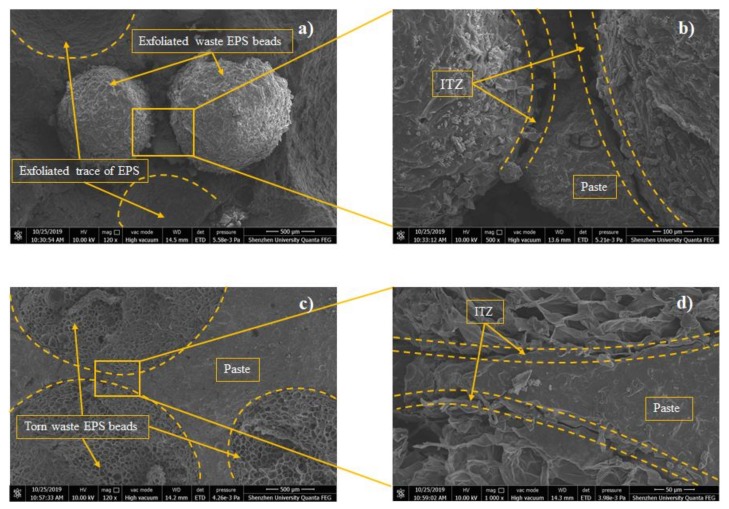
SEM images of microstructure morphology: (**a**) E8 at 120×, (**b**) E8 at 500×, (**c**) E8–G4 at 120×, and (**d**) E8–G4 at 1000× in 28 days.

The microstructures of the E8-G4 and E8 broken samples on the 28th day were observed under SEM, as shown in Figure 12. From Figure 12c,d, waste EPS beads have spatially porous and honeycomb internal structures, which explains the significant reduction of the composites’ density in Section 3.1. The special honeycomb internal structures of waste EPS beads are believed to improve the thermal insulation performance of AAMs effectively. A comparison of Figure 12a,c reveals a denser microstructure with fewer microcracks and micropores in composites with 0.04 wt.% rGO addition. Furthermore, the interface transition zone (ITZ) between the waste EPS beads/sand and pastes were focused on the addition to the AAMs. From Figure 12b,d, despite the hydrophobic properties of the waste EPS beads, the interfacial adherence between the waste EPS beads and the pastes in the sample containing rGO is tighter and more compact than that without rGO. In addition, according to the failure form in Figure 12a,c, most of the EPS was torn and destroyed with the expansion of cracks rather than peeled off from the paste when the sample with rGO was destroyed. The strong bond between the EPS and paste can be a plausible reason for the increased strength of the samples containing rGO.

### 3.5. Synergetic Analysis of Thermal Conductivity, Strength, and Cost

**Figure 13:**
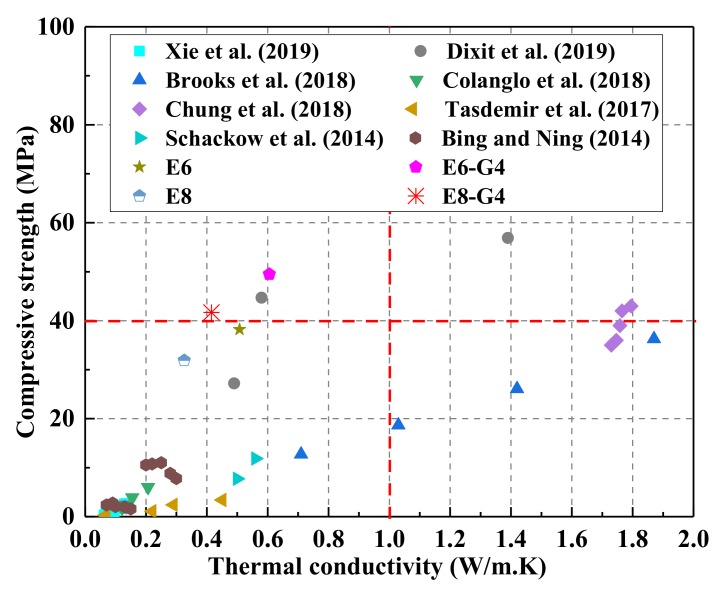
Contemporary works in the field of EPS insulating concrete.

The reduction in density of a composite generally leads to the decrease in its TC as well as compressive strength. The former reduces heat loss from the building envelope, but structural applications need greater compressive strengths. In this term, the compressive strength–thermal conductivity (CS–TC) ratio was utilized to provide a holistic and reasonable comparison of composites with potential functional and structural applications. The calculated values of the CS–TC ratio are listed in Table 7. As illustrated in the table, E8–G4 shows the highest CS–TC ratio (98.8) with CS of 41.5 MPa and TC of 0.42 W/m·K among the samples. Compared with E8 (with highest TC, CS–TC ratio of 95.3), the CS–TC ratio of E8-G4 significantly increases by 3.3%, respectively. It indicates that E8–G4 can obtain satisfactory CS under a low TC condition. Figure 13 compares the lightweight aggregates with contemporary works on insulating composites. In this figure, the results of this study are marked on the top-left corner of the data map, which strongly agree with the previous conclusions.

While most of the research studies involved traditional concrete with an expensive thermal insulating substitute, rGO-WEA contains economical waste EPS beads. Furthermore, the inclusion of waste EPS and rGO resulted in a more comprehensive performance with other composites in terms of thermal conductivity, strength, and cost. For a holistic and reasonable comparison with other composites, an efficiency index (EI) defined as compressive strength/(TC × Cost) was utilized, which is also listed in Table 7. The material costs were calculated, according to the market price of various ingredients in China and converted from China Yuan to USA dollar (USD).

**Figure 14:**
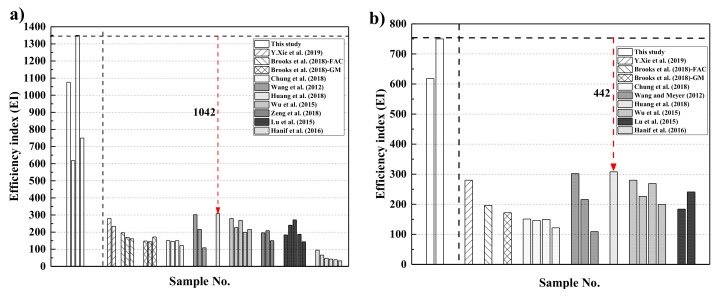
Efficiency indices for contemporary works with: (**a**) thermal conductivities less than 1.0 W/m·K and (**b**) thermal conductivities less than 1.0 W/m·K as well as satisfying minimum strength criteria (i.e., 40 MPa).

To validate the findings of this study, samples of previous researchers’ results were chosen for comparison, as in Figure 14. Figure 14a illustrates the samples limited to composites with TC values below 1.0 W/m·K. From Figure 14a, the maximum value of EI in this study, which is from E8 mixture, is 1357 and higher than that of the previous study. However, the compressive strength of E8 cannot meet the structural requirement standard (40 MPa). Figure 14b compares this study to the previous ones with compressive strengths greater than 40 MPa and TC values below 1.0 W/m·K. From Figure 14b, the EI of E8–G4 mixture is 442 higher than the highest value of the previous study. Therefore, rGO-WEA performs better than materials published in previous research studies and exhibits a fine balance among thermal conductivity, compressive strength, and cost. Furthermore, the integration of low TC, high compressive strength, and low cost of the rGO-WEA proposed in this study improves the energy efficiency of the building, which will be discussed in the next section.

### 3.6. Impact Assessment and Interpretation

#### 3.6.1. Environmental and Economic Impacts Assessment of Samples

Material production is a critical stage in the environmental and economic impacts assessment. The EE, ECO_2_e, and cost of each sample for the entire building, during the materials production stage, are accounted for and summarized in Table 8. According to the EI results in Section 3.5, the environmental and economic impacts of E8-G4 and E0 were chosen for further discussion. The results show that the EE, ECO_2_e, and cost of E8-G4 were 3382.0 MJ, 188.4 kg, and $131.8 per cube composites, respectively. The ECO_2_e of E8–G4 was significantly lower than that of E0 by 16.8%, while the EE and cost were higher, mainly due to the high energy requirement and expensiveness of rGO. The results demonstrate that rGO-WEA can reduce CO_2_ emissions during the proposed life-cycle boundary. This phenomenon can be attributed to the positive impact of recycling EPS on the environment. These benefits are particularly evident in developing countries, as their large populations, high demand, heavy industry, energy conservation, emission reduction, and other environmental protection approaches are more urgent.

#### 3.6.2. Energy Conservation Evaluation during the Operation Period

Building operation is another critical stage in the impact assessment. During this procedure, the energy conserved of the rGO-WEA application to an official building was evaluated by DesignBuilder software. This energy model considers the total heat balance of the internal and external building walling, the heat transferred through the building enclosures, and heat sources and sinks such as equipment, occupants, and lighting. To better compare the energy savings of different types of walls, three models were simulated for (i) traditional walls composed of three layers including 20-mm cement mortar, 200-mm concrete, and 20-mm cement mortar. The thermal conductivity of each layer was 1.50 W/m·K, 1.95 W/m·K, and 1.50 W/m·K, respectively, in accordance with the ASHRAE Standard 90.1 [68]. (ii) The middle layer was replaced with E0 (1.61 W/m·K) as a control sample and the other two layers were the same as the walls used by traditional walls, and (iii) the E8–G4 wall was composed of three layers. The middle layer is replaced with E8–G4 (0.42 W/m·K) and the other two layers are the same as the traditional walls.

**Figure 15:**
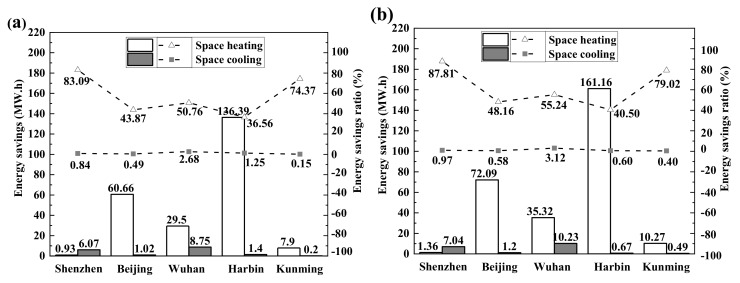
Space heating/cooling savings and saving ratios in different locations: (**a**) E8–G4 to E0 and (**b**) the E8–G4 to traditional concrete.

Figure 15 shows the annual energy savings of a building designed at five locations in China with the typical climatic features of country, which were obtained via energy simulation. Figure 15a shows the annual energy savings and saving ratio on space heating/cooling of cases E8–G4 to E0 in different locations. Figure 15b shows the same for cases from E8–G4 to the traditional concrete walls. E8-G4 saves plenty of space heating energy in the long winter season compared with traditional concrete both in Harbin and Beijing, which leads to 40.50% and 48.16% energy savings, respectively. The energy savings ratio of space heating in Shenzhen is as high as 87.81% of the low total energy consumption in the winter. Therefore, it does not contribute to this discourse.

Furthermore, E8–G4 reduces the cooling energy consumption of buildings in Shenzhen and Wuhan during the hot summer months with overall energy savings of 0.84% and 2.68% compared to E0. Note that the energy savings of space cooling is significantly lower than that of space heating. This low value is related to the larger indoor heat quantity during a long summer, where less heat loss means increased space cooling. However, the energy consumption of space cooling in Shenzhen and Wuhan is significantly higher. Hence, E8–G4 has potential in saving energy consumption. Given that the outdoor temperature of Kunming is relatively average during the summer and winter. Even ordinary walls stay at a comfortable temperature and the effect of rGO-WEA on energy savings is not enormous.

As seen in Figure 15b, compared to the traditional walls of ordinary concrete, the insulation property of E8-G4 plays an important role in building energy consumption. In Shenzhen, Beijing, Wuhan, Harbin, and Kunming, the total energy consumption (including heating and cooling) of the E8–G4 mixture against the traditional wall can be saved by 8.40 MW·h (1.15%), 73.29 MW·h (20.48%), 45.54 MW·h (11.63%), 161.83 MW·h (31.78%), and 10.76 MW·h (7.95%), respectively. For instance, the energy consumption (including heating and cooling) per annum of a building in Harbin can be saved by 161.83 MW·h, which is equal to the annual electricity use of 20 households in China [78]. Therefore, the full range of environmental benefits of rGO-WEA can be leveraged in regions with long and cold winters or hot summers, as it considerably reduces the consumption of building energy.

## 4. Conclusions

In this study, waste EPS beads, rGO, and AAMs were synthesized to successfully develop a novel structural and functional integrated materials (rGO-WEA). The mechanical strength, microstructure, density, and TC of rGO-WEA were investigated. In addition, LCA and BIM tools were integrated to analyze the environmental and economic sustainability of rGO-WEA. The following conclusions can be drawn from the results.

(1) The wet and dry densities of rGO-WEA decreased with the increase of waste EPS beads. The lower the density is, the lower the thermal conductivity is. The addition of rGO slightly increased the density of rGO-WEA owning more reaction products caused by rGO. However, the rGO content adopted in this study was relatively low. Thus, the increase in wet and dry densities of rGO-WEA were not significant.
(2) The introduction of rGO into AAMs is an effective method to improve the ITZ between waste EPS beads and paste and counter the degradation of compressive strength caused by the addition of waste EPS beads. Even with 80 vol.% EPS replacement, the compressive strength measured in E8–G4 was 41.5 MPa, which meets the minimum 28th day compressive strength of the ACI 213R-03 standard for structural applications.
(3) The thermal conductivity of E8-G4 at the 28th day reduced by 76.4% and 74% compared with E0–G4 and E0, respectively, because of lowering of rGO-WEA density with the increased content of waste EPS beads in the AAMs matrix. Therefore, rGO-WEA has the potential to accelerate waste EPS recycling, and promote sustainable structural and functional cementitious composites for the construction industry.
(4) The EI value of E8–G4 is 750 and increases by 442 from the previous highest value. This indicates that rGO-WEA can help strike a fine balance between the compressive strength, thermal conductivity, and cost as a structurally and functionally integrated material.
(5) The results of the coupled LCA–BIM environmental evaluation approve the rGO-WEA in this study, which can conserve large amounts of energy and reduce CO_2_ emissions in the building structural application, especially, in developing countries. The ECO_2_e of E8–G4 was reduced by 16.8% when compared with E0. Furthermore, in cold areas such as Harbin, using E8–G4 in building envelopes can save space heating energy consumption by 40.50% and, in hot areas, such as Wuhan, can save space cooling energy consumption by 3.12%, when compared with traditional concrete.

## Figures and Tables

**Table 1:** Contemporary works on insulating concrete with EPS beads.

Authors	EPS to Aggregate Ratio (%)	Thermal Conductivity (W/m·K)	Density (kg/m^3^)	28th-Day Compressive Strength (MPa)
Xie et al. (2019)	20–40	0.129–0.139	300–600	1.91–2.47
Dixit et al. (2019)	0–45	0.49–2.14	1463–2301	27.2–149.8
Brooks et al. (2018)	0–28.5	0.71–2.5	1300–2084	12.4–43.6
Colangelo et al. (2018)	65–72.5	0.13–0.17	545–750	1.8–2.4
Chung et al. (2018)	0–70	1.730–1.790	1698–2093	35–43
Tasdemir et al. (2017)	0–60	0.23–0.45	300–1600	0.3–18.7
Ning and Bing (2014)	0–46.5	-	1124–2084	7.5–62.6
Schackow et al. (2014)	55–65	0.50–0.56	1110–1250	7.74–11.85
Bing and Ning (2013)	0–20	0.2–0.3	805–1100	7.79–10.56
Yi et al. (2012)	15–25	-	1720–2060	11.22–20.77

**Table 2:** Chemical compositions of GGBFS and SF.

Component (wt.%)	CaO	SiO_2_	Al_2_O_3_	MgO	TiO_2_	Fe_2_O_3_	MnO	Na_2_O	K_2_O	[OH]	LOI
GGBFS	38.95	35.24	14.93	7.36	0.57	0.20	0.69	0.33	0.38	0.58	0.77
SF	0.49	93.26	1.29	0.95	-	1.97	-	0.42	1.05	-	0.57

**Table 3:** Physical characteristics of sand, waste EPS, and standard requirements.

Fine Aggregates	Sand	EPS	Standard Requirement (GB/T18046)
Thermal conductivity (W/m·K)	0.5	0.042	≤1.0
Apparent density (g/cm^3^)	2.63	0.028	≤3.0
Mass density (g/cm^3^)	1.49	0.018	≤2.0
Compact density (g/cm^3^)	1.58	-	≤2.0

**Table 4:** Mixture proportions of alkali-activated slag mortar in this study.

Sample No.	GGBFS (g)	EPS (g)	Sand (g)	Water (g)	NaOH (g)	LSS (g)	SF (g)	rGO (g)	rGO/Binder (wt.%)
E0	1080	0	1800	209.6	25.6	156.7	120	0	0
E0–G4	1080	0	1800	209.6	25.6	156.7	120	0.48	0.04
E6	1080	11.5	720	209.6	25.6	156.7	120	0	0
E6–G4	1080	11.5	720	209.6	25.6	156.7	120	0.48	0.04
E8	1080	15.4	360	209.6	25.6	156.7	120	0	0
E8–G4	1080	15.4	360	209.6	25.6	156.7	120	0.48	0.04

**Table 5:** Thermo–physical properties of materials used in the building model.

Building Materials	Density (kg/m^3^)	Thermal Conductivity (W/m·K)
Concrete*	2300	1.95
Mortar*	1900	1.50
E0	2405	1.61
E8–G4	1498	0.42

* Data sources of concrete and mortar according to ASHRAE Standard (2013).

**Table 6:** Input parameters for calculating EE and ECO_2_e costs.

Material	EE* (MJ/kg)	ECO_2_e* (kg/kg)	Cost (USD/ton)
Waste EPS	−0.3409	−3.181	213.17
Natural/river sand	0.0148	0.0014	8.53
GGBFS	1.6	0.083	44.05
LSS	15.98	1.237	92.37
NaOH	20.55	1.414	284.22
Water	0.0025	0.0002	1
SF	0.018	0.014	200
rGO	33.5007	0.367	1.3 × 10^5^

* Data sources of EE and ECO_2_e are from eBalance. Costs are current market values.

**Table 7:** Ratios of compressive strength to thermal conductivity and EI calculations of the composites.

Mix ID	TC (W/m·K)	CS (MPa)	Cost (USD/m^3^)	CS–TC Ratio	EI
E0	1.61	88.6	75.8	55.0	726
E0–G4	1.77	114.5	137.6	64.7	470
E6	0.50	38.2	71.1	76.4	1076
E6–G4	0.61	49.4	132.8	81.0	618
E8	0.33	31.9	70.2	95.3	1357
E8–G4	0.42	41.5	131.7	98.8	750

**Table 8:** Values of EE, ECO_2_e, and production cost calculated for the various samples.

Mix ID	EE*	ECO_2_e*	Cost*
	(MJ/m^3^)	(kgCO_2_e/m^3^)	(USD/m^3^)
E0	3389.7	229.3	75.8
E0–G4	3401.1	229.4	137.6
E6	3375.6	202.7	71.0
E6–G4	3387.0	202.8	132.8
E8	3370.9	190.6	70.3
E8–G4	3382.3	190.7	131.7

* Data sources of EE and ECO_2_e are from eBalance. Costs are current market values.
